# Elucidating the clonal relationship of esophageal second primary tumors in patients with laryngeal squamous cell carcinoma

**DOI:** 10.1186/s13027-023-00558-z

**Published:** 2023-11-28

**Authors:** Meixuan Wan, Xinxin Yang, Lin He, Hongxue Meng

**Affiliations:** 1 Department of Pathology, Harbin Medical University Cancer Hospital, Harbin, 150081 China; 2 Precision Medicine Center, Harbin Medical University Cancer Hospital, Harbin, 150081 China; 3 Department of Stomatology, Heilongjiang Province Hospital, Harbin, 150081 China

**Keywords:** Laryngeal squamous cell carcinoma, Esophageal second primary tumor, Clone, Field cancerization, Epigenetics, Tumor microenvironment

## Abstract

Laryngeal cancer ranks as the second most prevalent upper airway malignancy, following Lung cancer. Although some progress has been made in managing laryngeal cancer, the 5-year survival rate is disappointing. The gradual increase in the incidence of second primary tumors (SPTs) plays a crucial role in determining survival outcomes during long-term follow-up, and the esophagus was the most common site with a worse prognosis. In clinical practice, the treatment of esophageal second primary tumors (ESPT) in patients with laryngeal squamous cell carcinoma (LSCC) has always been challenging. For patients with synchronous tumors, several treatment modalities, such as radiotherapy, chemotherapy and potentially curative surgery are necessary but are typically poorly tolerated. Secondary cancer therapy options for metachronous patients are always constrained by index cancer treatment indications. Therefore, understanding the clonal origin of the second primary tumor may be an important issue in the treatment of patients. LSCC cells demonstrate genetic instability because of two distinct aetiologies (human papillomavirus (HPV)-negative and HPV-positive) disease. Various etiologies exhibit distinct oncogenic mechanisms, which subsequently impact the tissue microenvironment. The condition of the tissue microenvironment plays a crucial role in determining the destiny and clonal makeup of mutant cells during the initial stages of tumorigenesis. This review focuses on the genetic advances of LSCC, the current research status of SPT, and the influence of key carcinogenesis of HPV-positive and HPV-negative LSCC on clonal evolution of ESPT cells. The objective is to gain a comprehensive understanding of the molecular basis underlying the clonal origins of SPT, thereby offering novel perspectives for future investigations in this field.

## Background

Globally, the number of incidence cases of laryngeal cancer climbed from 132 700 in 1990 to 210 600 in 2017 [1]. In 2018, it was anticipated that 177 000 instances of laryngeal cancer and 95 000 related deaths occurred worldwide [2]. Lin et al. [3] predict the age-standardized incidence of laryngeal cancer from 2019 to 2035, and despite its projected decline, the estimated absolute numbers are still expected to rise, and laryngeal cancer remains a serious health issue globally. Additionally, a growing number of SPTs have been diagnosed as a result of improved diagnostic techniques, which have the potential to progress into various types of cancer, including multicentric cancers affecting the lung, esophagus, and head and neck. Notably, in patients with head and neck cancer, SPTs contribute to a considerable proportion of mortality, accounting for approximately one-fourth to one-third of deaths in this patient population [4]. The prognosis of esophageal cancer is poorer in these SPTs than in other sites of the upper gastrointestinal tract [5]. Furthermore, ESPT is characterized by flat lesions that are readily missed with high-resolution white light endoscopy [6]. Higher prevalence of ESPT, and it has been discovered that the incidence of ESPT in patients with head and neck squamous cell carcinoma (HNSCC) in Asia reached 17.7% (358 of 2627, 95% CI 12.7–22.7), of which 3.4% (19 of 474, 95% CI 1.8–5.4) are ESPT in patients with LSCC [7]. Furthermore, it was observed that patients diagnosed with HNSCC accompanied by SPTs exhibited a significantly lower 15-year survival rate compared to those without SPTs, with rates of 22% and 54% respectively. Additionally, patients with SPTs experienced a particularly unfavorable prognosis, as evidenced by a 5-year survival rate of only 6% for ESPT, in contrast to the 25% survival rate observed in patients with all types of SPTs [8]. Another study corroborated these findings, demonstrating that patients with LSCC who developed SPTs had lower 5-year (68% vs. 76%) and 10-year (26% vs. 57%) overall survival rates, with statistical significance (*P* = 0.003) [9]. Consequently, the presence of ESPT significantly impacts the survival outcomes of patients diagnosed with LSCC.

Successful treatment of second primary tumors in patients with LSCC is a matter of clinical significance. Another aspect extensively debated in scholarly literature is the clonality of second and index tumors. Cui et al. [10] used loss of heterozygosity (LOH) analysis to reveal that recurrent LSCC is of monoclonal origin, but Sunpaweravong et al. [11] used a genome-wide SNP array to find a highly inconsistent LOH pattern between LSCC and ESPT, thereby suggesting that ESPT belongs to an independent clonal evolution. Presently, the clonal relationship between LSCC and ESPT has not yet been clearly stated. Furthermore, apart from the evident genomic disparities between HPV-positive and HPV-negative LSCC (Table 1), disparities in crucial oncogenic mechanisms should be taken into account. Molecular modifications play a significant role in the emergence and evolution of tumor cell clones. The pathogenesis of LSCC is different, and the occurrence and development of the disease are also different. Consequently, it is imperative to discuss the clonal relationship between LSCC and ESPT by considering the molecular basis of their distinct etiologies.

**Table 1:** Major differences between LSCC by HPV status

	HPV-positive LSCC	HPV-negative LSCC
Risk factors	High-risk sexual practices	Tobacco and alcohol
P53 pathway disturbances	Degradation of wt p53 by E6	TP53 mutations, 17p13LOH
Mutational burden	Low	High
Rb pathway disturbances	Degradation of wt Rb by E7	p16INK4A-promoter hypermethylation, 9p21 LOH
immune microenvironment	Hot	Cold
Factors mediating cellular transformation	E6 and E7 hr-HPV oncoproteins Genomic rearrangements induced by viral genome integration	Hypermutational status and chromosomal instability induced mainly by alcohol and tobacco carcinogens
Prognosis: 5-year survival rates	More favorable	Less favorable
Relative responsiveness to chemoradiation	Better	Worse
Relative prognosis	Improved	Worse

## Genetic progression of LSCC

Squamous cell carcinomas are one of the most common solid cancers involving many anatomical sites in humans, exhibiting a propensity for metastasis and dissemination and constituting a leading cause of mortality worldwide [12]. Histologically, the progression of invasive squamous cell carcinoma follows a sequential pattern, initiating with the proliferation of epithelial cells, succeeded by a range of dysplasia from mild to severe, then progressing to in situ carcinoma, and ultimately culminating in invasive carcinoma [13]. The formation of tumors is widely acknowledged as a highly intricate process that encompasses numerous genes. Tumor suppressor genes and oncogenes are altered genetically and epigenetically during the transition from normal cells to cancer cells [14]. Additionally, tumors usually occur in the precancerous areas of genetically altered cells. The continued presence of these tumor fields following therapy presents a significant difficulty, as it heightens the likelihood of second primary tumors and local recurrence, both of which are significant contributors to mortality [15]. Microsatellite instability is the basis of a unique tumorigenic pathway. Recent investigations have revealed that diminished expression of mismatch DNA repair genes heightens the susceptibility to microsatellite instability, a phenomenon frequently observed in head and neck cancer [16]. LSCC cells demonstrate genetic instability and commonly manifest a diverse array of chromosomal modifications, including translocations, amplifications, and deletions. Although LOH analysis and array comparative genomic hybridization (aCGH) have identified numerous chromosomal regions, the majority of genes within these regions remain unidentified.

Frequent allelic deletions were observed at chromosomal positions 3p, 4q, 9p (p16^INK4a^), 11q, 13q (Rb), 14q and 17p (TP53) in LSCC patients [10]. Additionally, LOH analysis provides valuable insights into the identification and characterization of tumor suppressor genes and their cloning process [17]. It is worth noting that microsatellite DNA can be inherited across generations. The utilization of LOH analysis can be beneficial in distinguishing between synchronous primary ovarian and endometrial malignancies and single metastatic tumors [18]. Zhu et al. [19] employed LOH analysis to show that two nearby hepatocellular carcinomas looked to be intrahepatic metastases of two original tumors. Recently, Cui et al. [10] used LOH to demonstrate that primary and recurrent LSCC in the same patient have the same allelic deletion pattern and finally concluded that both types of tumors originated from the same clone. LOH analysis provides important clues to the clonal origin of tumor cells.

## Current research status of SPT

**Fig. 1:**
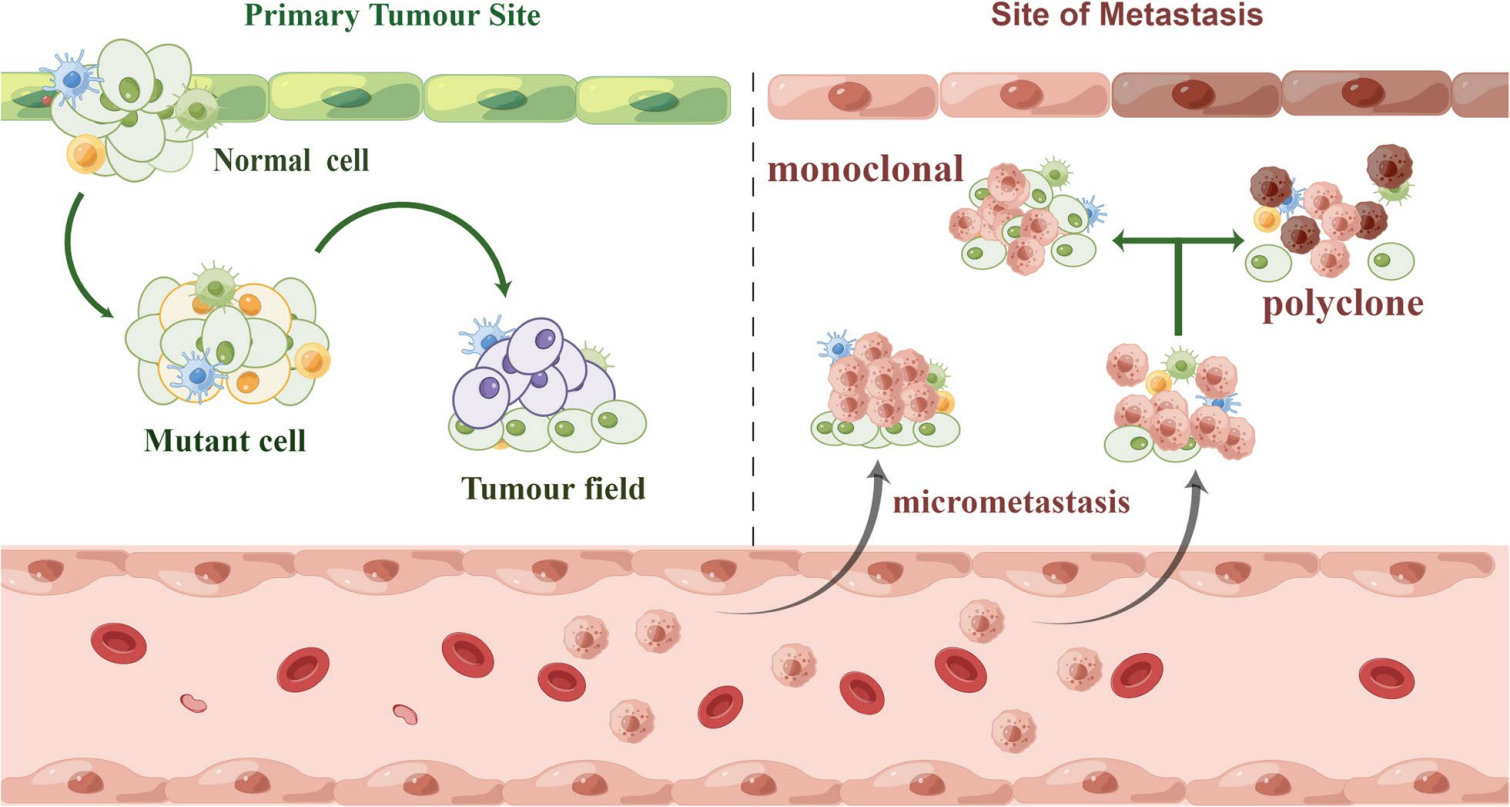
The main mechanisms by which SPT occurs. Normal cells receive one (or more) genetic hits and produce a daughter cell with genetic alterations that gradually replace normal epithelial cells to form a tumor field. When the tumor field expands at the expense of normal epithelial cells, additional genetic alterations occur, prompting the tumor field to develop into obvious cancer and metastasize or the cancer cells to form a second tumor by submucosal spread or intraepithelial migration or to form a polyclone with additional induction

Most doctors currently adhere to the criteria established by Warren et al. (1932) to define SPT: (a) each tumor must exhibit malignancy, (b) each tumor must be independent, and (c) the possibility of metastasis from one tumor to another must be ruled out. In cases of similar histotypes, SPT was considered when the second tumor was more than 2 cm away from the index tumor or occurred more than three years after the index tumor. However, no agreement was reached on these matters. SPT can be divided into two groups: simultaneous SPT, where both are diagnosed that occur within six months of one another or at the same time; for metachronous SPT, where both are diagnosed more than six months apart. Most SPT is heterochronic and is detected at follow-up after treatment of the first tumor [20]. In fact, SPT, as the name suggests, these tumors develop independently of the primary tumor. However, genetic studies have revealed that in a certain proportion of cases, the two have a certain clonal relationship [21]. It was later suggested that the term “SPT” be assigned to a second tumor that develops completely independently. In cases where both tumors share the same clonal origin, they are referred to as “second field tumors” (SFT). Consequently, there exist three primary mechanisms through which SPT manifests: (1) Micrometastasis (cloning); (2) Arising from a shared oncogenic field (SFT-partial cloning); (3) Independent occurrences (true SPT-non-cloning) [22] (Fig. 1). It is imperative to establish this differentiation as the management of SPT currently poses a formidable clinical challenge.

An increasing body of literature has emerged regarding SPTs, with a predominant emphasis on investigating their incidence, treatment, and prognosis. However, limited attention has been given to exploring the clonal relationship between primary tumors and SPTs. Various laboratory techniques, such as karyotyping, cytogenetic methods, TP53 mutations, X-chromosome inactivation, aCGH, LOH, and more recently, whole-genome or exome next-generation sequencing, have been proposed as means to evaluate this clonal relationship [23]. But mutations are cumulative, tend to change during cancer progression, and lack specificity, so the reliability of molecular techniques is in doubt. For instance, LOH analysis can only indicate the proliferation of monoclonal clones when multiple and consistent LOH is observed. Furthermore, the use of aCGH and X-chromosome inactivation is not suitable for routine clinical practice, aCGH is costly and time-consuming, while X-chromosome inactivation is only applicable to female samples [24, 25]. mtDNA is considered to be an effective tool for evaluating the clonal relationship between primary tumors and SPTs, but it also has some limitations [26]. Clonal evolution is a highly intricate phenomenon, and genomics serves as a potent tool for elucidating tumor evolution by enabling the assessment of clonal evolution across spatial and temporal dimensions [27]. One study used integrated multi-omics methodologies such as transcriptomics, whole genome sequencing, and immune cell receptor sequencing to investigate the impact of the spatial organization of the immunological microenvironment on the evolution of ovarian cancer clones [28]. Future research should focus on developing simpler and more precise approaches for determining clonal origins, such as multi-omics strategies, in order to better understand the evolutionary mechanisms of cancer.

## Hypothesis of field cancerization

**Fig. 2:**
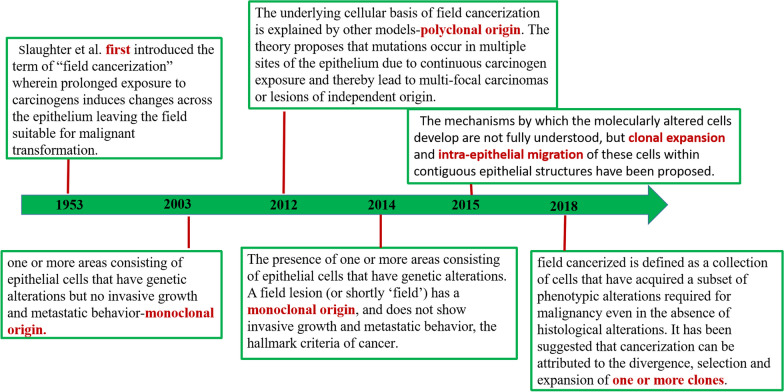
Timeline: Historical definition of a field cancerized (Cellular basis)

Clinicopathological evidence serves as the established criterion for distinguishing distant metastases from SPT, but this approach is relatively subjective [29]. In 1953, Slaughter et al. introduced the concept of “field cancerization” to explain the pathogenesis of SPT, and it has since undergone further development (Fig. 2). The term “field cancerization” has been extended to encompass various other organs, including the esophagus, oropharynx, stomach, larynx, lungs, anus, colon, cervix, skin, and bladder. Therefore, this description effectively elucidates the theory of multifocal tumor origin. At present, there are two views on the “field cancerization” hypothesis: the first supports a monoclonal origin since “jumping lesions” are always present. That is, the tumor cells expand from the primary site to nearby sites. Although the extent of clonal expansion is unknown, some observations suggest that this process may result in tumor cells extending well beyond the microscopic boundaries of the tumor mass. That surface migration of clonal cells may be widespread even without obvious histopathological changes in the malignancy [30]. Specific genetic alterations occur throughout the airways when exposed to carcinogens, with one genetically altered cell producing a proliferating clone that grows into an expanding area of cancer and gradually replaces the normal mucosa. In this genetically altered area, accumulating other independent genetic alterations provides an additional growth advantage to the cell subpopulation. In the process, eventually, a subclone evolves into aggressive cancer. This is an ongoing evolutionary process, and although they are genetically distinct, they share a common origin [21]. Conversely, the other explanation posits that mutations arise in many epithelial locations as a result of ongoing carcinogen exposure, resulting in multifocal carcinomas or lesions of independent origin [31]. As a result, the interpretation of the clonal relationship between SPT and index tumors based solely on “field cancerization” is divergent and requires further investigation.

## The impact of clonal evolution

### Key gene mutations

In the absence of HPV, an increased risk of SPTs was found [32]. Consuming alcohol and smoking are the two most significant risk factors for HPV-negative LSCC and esophageal squamous cell carcinoma (ESCC). These carcinogens can directly damage and mutate DNA, increasing mutational burden, intercellular heterogeneity, and driving mutations [33]. TP53 tumor suppressor mutations have been shown to be the most common genetic variant and stable, well-distributed point mutation (exons 5–8) in LSCC, rendering this mutation a significant predictor of treatment outcome and valuable for clonality assessment [34]. Histopathological studies of ESPT in patients with HPV-negative LSCC have identified small TP53 mutated plaques (less than 200 cells in diameter) that precede the expansion of cancer [35]. But Chen et al. [36] conducted mutation analysis of the TP53 core binding domain and found no clonal relationship between LSCC and ESPT. The mutation pattern observed in the *P53* gene exhibits considerable variability, suggesting a polyclonal nature of transformation. It is important to note that tumor development is a multifaceted process, necessitating additional genetic alterations and a large number of stromal cells (inflammatory cells, immune cells, etc.) for the transformation of plaques into extended fields. *P53* gene mutations and sustained loss of cell differentiation, along with the entry of cells with damaged DNA into the S phase, lead to altered genetic characteristics and chromosomal aberrations, promoting tumor development [37]. Furthermore, P53 is essential for the repair of DNA damage and the initiation of apoptosis. Consequently, the mutation of the *TP53* gene observed in patients with LSCC, attributed to smoking, hinders the timely repair of DNA damage, resulting in the continuous proliferation of cells carrying genetic abnormalities. This uncontrolled proliferation ultimately leads to the hypermutation of tumor cells [38, 39]. While the cancer stem cell hypothesis traditionally opposes the notion of polyclonal tumor origins, the interaction among numerous pluripotent stem cells presents a potential mechanism for polyclonal tumors origins [40].

Some studies have found that alcohol-induced LSCC and ESPT also have an independent origin, particularly in individuals with alcohol-induced facial flushing [41]. The alcoholic flush response is associated with a deficiency of acetaldehyde dehydrogenase (ALDH2), which results from excessive accumulation of acetaldehyde (AD). There is growing evidence that people who lack ALDH2 are at much higher risk of developing LSCC and ESCC from alcohol consumption than those with fully functional ALDH2, while ALDH2 × 1/2 heterozygotes are also at a heightened risk of developing LSCC and ESCC [42]. When significant quantities of AD accumulate in saliva, it results in direct contact between AD and the upper airway mucosa, potentially leading to genetic mutations [43]. AD has the ability to bind to DNA, forming stable DNA adducts that initiate DNA damage. Moreover, if these AD-induced DNA adducts manage to evade cellular repair mechanisms and persist, they can ultimately give rise to coding errors and genomic instability [44]. Genomic instability can have an impact on chromosome structure, chromosome number, and DNA sequence, and in certain cases, it can compromise genomic integrity across multiple levels simultaneously. In extreme cases, resulting in a huge number of mutations in tumor cells, the effects of genetic drift (random loss or immobilization of genotypes) can be amplified so that neutral or even harmful mutants can be retained during enlargement, leading to clonal diversity [45]. Therefore, there is a preference for independent clonal origin between HPV-negative LSCC and ESPT.

HPV-positive tumors behave biologically differently from HPV-negative tumors, thereby influencing the pathophysiology of the disease [46]. The early occurrence of HPV infection is observed in both LSCC and ESCC, with HPV16 being the predominant pathogenic subtype [47, 48]. HPV is a circular, double-stranded DNA virus with a genome size of approximately 8 kb, lacking an envelope, and belonging to the papillomavirus family. This epitheliophilic virus specifically targets basal cells in stratified epithelial tissues in mucosal or cutaneous regions. Infection is initiated when the virus reaches the basal cells and replicates viral DNA via the cellular DNA replication machinery, producing a small number of copies of the circulating virus. The viral genome is organized into three sections: the early genetic region (E), the late genetic region (L), and the long regulatory region (LCR) that connects the two. The late region encodes L1 and L2 proteins, which are responsible for encoding the primary and minor viral capsid proteins, respectively, the early region codes for E1-E5 genes primarily involved in viral genome replication and transcription [49]. It is essential for both E6 and E7 genes to be present in order to induce oncogenic transformation in host cells. Furthermore, the expression of E6 and E7 is typically elevated in advanced precancerous stages, leading to the transformation of infection [50]. The integration of viral DNA into the host cell genome is a crucial step in the progression of HPV to cancer [51].The whole cellular genome, including both gene-rich and gene-poor areas, is subject to viral integration. Initial investigations into viral integration causing cervical lesions indicate that this process is stochastic and may exhibit a preference for microhomologous regions, areas with strong transcriptional activity, common weak sites, or areas near microRNAs (miRNAs) [52]. Integration into or near genes can result in alterations in gene expression through various mechanisms, including the formation of viral-cellular fusion transcripts. However, the precise mechanism remains unclear [53]. Parfenov et al. [54] observed an elevated somatic DNA copy number in the integrated region and reported that HPV viral integration disrupts genetics through multiple crucial pathways, such as the loss of tumor suppressor function, increased expression of oncogenes, and rearrangement of gene expression. It is rare for the *P53* gene to be altered in HPV-positive LSCC, which is usually eliminated by E6 [49]. The E6 protein, in a protein-dependent manner, binds to the core region of the *P53* protein, resulting in the formation of the E6/E6AP complex. This complex facilitates the degradation of *P53* through a ubiquitin-dependent pathway, thereby promoting tumor progression [55]. Additionally, the E7 protein interacts with various cell cycle regulatory proteins, influencing their levels and/or cellular activity. One such interaction involves the high affinity of E7 for pRb, which leads to feedback upregulation of p16INK4A [56]. This upregulation inhibits the interaction between pRb and the transcription factor E2F, which is responsible for controlling cell cycle G1/S changes. Consequently, this disruption of the cell cycle promotes oncogenic transformation and clonal amplification [57]. Moreover, tumor growth exhibits a clonal evolutionary trajectory characterized by sequential clonal amplification, genetic diversification, and clonal selection. The stochastic nature of viral integration sites adds to tumor cell genetic diversity, and integration sites are passed down through clonal amplification [58]. One study revealed the clonal origin of bilateral HPV16-positive tonsillar tumors by viral integration analysis, which finally supported the monoclonal hypothesis [59]. Therefore, based on DNA integration, a key step in HPV viral carcinogenesis, it was found that HPV-positive LSCC secondary to ESPT is likely to be the result of clonal amplification. However, one case study suggests that there may be no clonal relationship between the LSCC and ESPT, and the exact mechanism needs to be further elucidated [60].

### Epigenetic changes

**Fig. 3:**
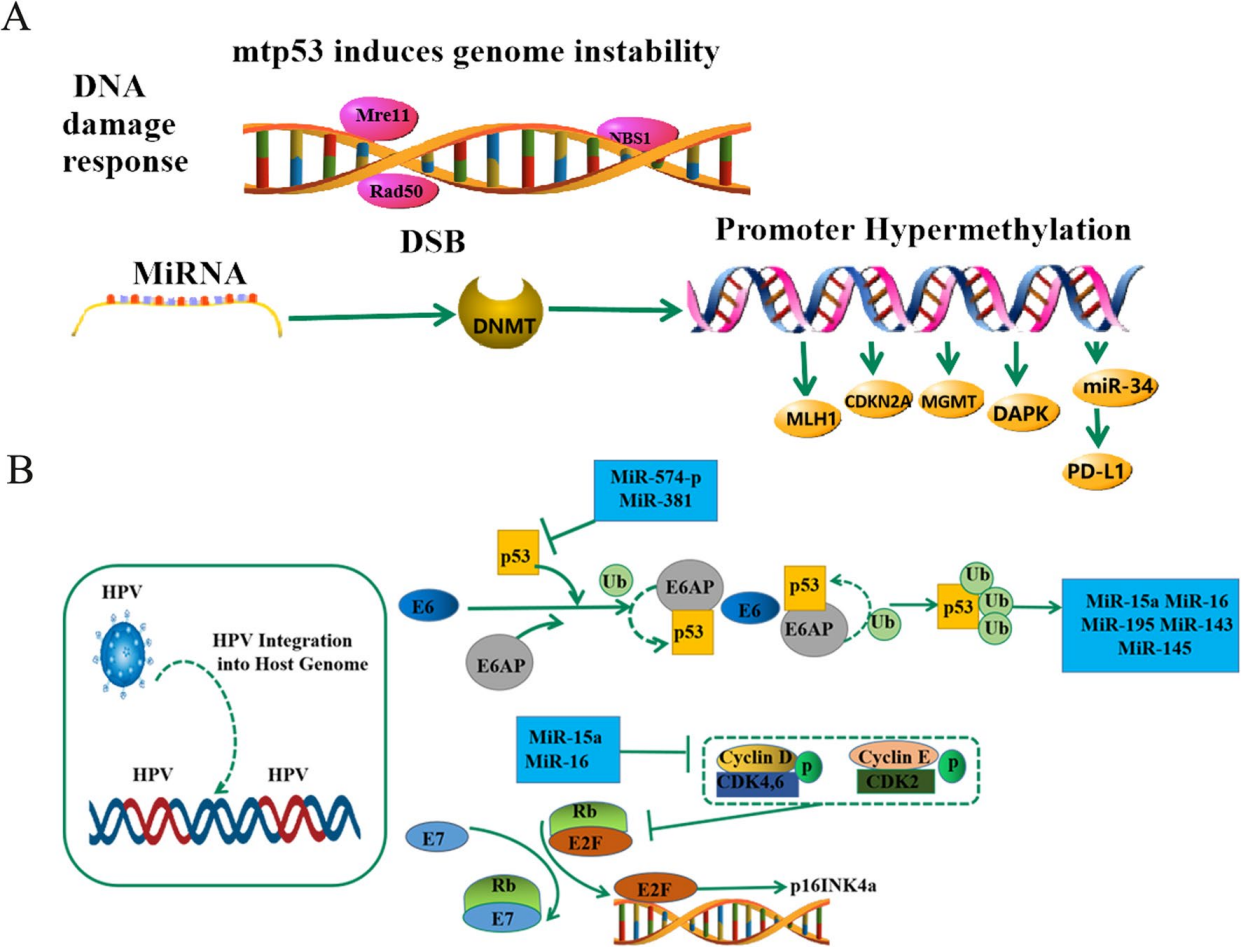
Key oncogenic mechanisms in HPV-negative and HPV-positive laryngeal cancers. A: Smoking and alcohol consumption cause mutations in the TP53 gene and affect DNA methyltransferase (DNMT)-dependent regulation of miRNA expression, leading to tumor transformation. B: By engaging the ubiquitin-protein ligase E3A (E6AP), E6 binds with p53 and promotes its degradation. Ubiquitinated TP53 also causes changes in the expression of miR-16, miR-15a, miR-143, miR-145, and miR-195. Cell cycle protein D1 expression begins when cell growth signaling occurs. Cell cycle protein E then activates CDK2, phosphorylates RB, releases E2F, and initiates cell cycle entry gene transcription

Gene expression can be altered through epigenetic modification without altering nucleotide sequences. These changes can impede apoptosis, disrupt the cell cycle, facilitate the growth of precancerous cells, and result in the expansion of clonal cell populations that are susceptible to new carcinogens. Over time, these alterations can accumulate carcinogenic events and contribute to the development of secondary primary tumors [61]. Additionally, epigenetic modifications like histone alterations, DNA methylation, chromatin remodeling, and microRNA can serve as potential indicators of cancer growth and progression [62]. Altered DNA methylation patterns are commonly observed in LSCC. These patterns typically involve the hypermethylation of tumor suppressor oncogenes and the hypomethylation and transcriptional deletion of proto-oncogenes, followed by transcriptional reactivation [63]. In HPV-negative LSCC patients, genes such as CDKN2A, MGMT, MLH1, and DAPK are frequently methylated, resulting in the inhibition of gene transcription and gene silencing [64]. According to the principles of phylogenetic tree analysis, mutations in TP53 and copy number alterations at 3q (contains SOX2), 9p (contains CDKN2A), 11q (contains CCND1), and 2q (contains NFE2L2) are considered to be backbone variants. During the progression from intraepithelial neoplasia to malignancy, certain genes exhibit clonal dominance, resulting in the clonal diversity of tumor cells [65]. Additionally, tobacco smoke and alcohol can modify the cellular chromatin through histone modification and impact gene transcriptions [66]. DNA methylation status plays a crucial role in gene regulation and is closely associated with histone modifications. Active gene expression is linked to histone H3 lysine 9 (H3K9) acetylation and histone H3 lysine 4 (H3K4) biomethylation [67]. Furthermore, it has been observed that smoking and alcohol consumption have the potential to induce genetic damage in miRNA genes, particularly in regions characterized by single nucleotide polymorphisms. These regions are closely linked to the regulation of the *P53* gene [68]. In the case of mutant TP53, there is an upregulation of programmed cell death ligand 1 (PD-L1) due to the modulation of miR-34 activity. Conversely, in wild-type TP53 tumor cells, DNA damage leads to an increase in miR-34 expression, which subsequently interacts with the 3’-untranslated region of PD-L1 and suppresses its protein expression. This TP53/miR-34/PD-L1 pathway highlights the significant disparity in PD-L1 production between TP53 mutant tumors and wild-type tumors [69]. It is hypothesized that the higher levels of PD-L1 exhibited by HPV-negative LSCC patients are likely due to the synchronous dual primary LSCC/ESPT with increased TP53 of the mutant type (Fig. 3A).

The degree of CpG island DNA methylation in HPV-positive LSCC was found to be significantly higher compared to HPV-negative LSCC. The most researched DNA methyltransferases are DNMT3a, DNMT3b, and DNMT1. It has been observed that the overexpression of DNMT1, DNMT3a, and DNMT3b is induced by the viral proteins E6 and E7, resulting in overall DNA hypermethylation [70]. This epigenetic alteration plays a role in the pathogenesis of respiratory papillomatosis recurrences, with some cases showing clonal changes in the progression of recurrences. Additionally, similar epigenetic events have been identified in two cases of HPV-infected laryngeal benign lesions that progressed to squamous cell carcinomas [71]. In the comparison between HPV-positive LSCC and cervical squamous cell carcinoma, there was an observed increase in the expression of miR-15a, miR-16, miR-195, miR-143, and miR-145. Significant overlap of these differentially expressed microRNAs was also found, suggesting that HPV-dependent microRNA expression disturbances are often present regardless of the anatomical location of the tumor [72]. Further investigations suggest that this miRNA expression may be directly influenced by viral E6 and E7 oncoproteins [73]. According to this research, viral integration events commonly cause host chromatin alterations, which further influence the carcinogenic process of HPV-positive malignancies [74]. The papillomavirus genome is connected with transcriptionally active host chromatin regions throughout the viral life cycle to enhance viral replication, transcription, DNA amplification, and persistence [75]. The primary drivers of these viral oncogenes are the oncoproteins E6 and E7, which not only induce infection but also contribute to epigenetic alterations associated with malignant transformation. These oncoproteins interact with cellular chaperones involved in the interdependent viral and cellular cycles within complexly differentiated epithelia [76]. Overall, HPV-positive LSCC exhibits susceptibility to DNA methylation, and its oncogenic mechanism primarily relies on the oncoproteins E6 and E7. Consequently, it is probable that LSCC and ESPT originate from a shared source and demonstrate shared genetic variations (Fig. 3B).

### Tumor microenvironment

The process of cancer cell division, resulting in the emergence of tumor cells, induces notable molecular, cellular, and physical modifications in the surrounding tissues, thereby establishing a tumor microenvironment (TME). This interplay between cancer cells (seeds) and the microenvironment (soil) facilitates the progression of tumor growth [77]. TME is not totally homogeneous, as different regions of the tumor may exhibit diverse blood densities, lymphovascular networks, immune infiltrating cell populations, and extracellular matrix compositions. External oncogenic factors can not only modulate cell signaling to directly cause phenotypic diversity in tumor cells but can also act as selection pressures, leading to regional heterogeneity and supporting the cloning of cells that proliferate efficiently in the context of a given microenvironment [78]. For example, tumor cells with the same genotype within a clone can exhibit varied behavior in response to alterations in the microenvironment (such as hypoxia, immune monitoring, and additional extrinsic variables), leading to intratumor heterogeneity [79]. Likewise, microenvironmental factors, such as the proximity of cancer cells to cancer-associated fibroblasts or hypoxia, can affect the “quiescence” of cancer cells, causing the cells to exhibit more or less stem cell-like behavior. In addition, it is becoming clear that host-tumor reactivity, as mediated by immune cells in the tumor microenvironment, is important for tumor formation [80]. The immune cell-mediated host-tumor reactivity within the TME establishes a foundation for the development of clonal tumors.

**Fig. 4:**
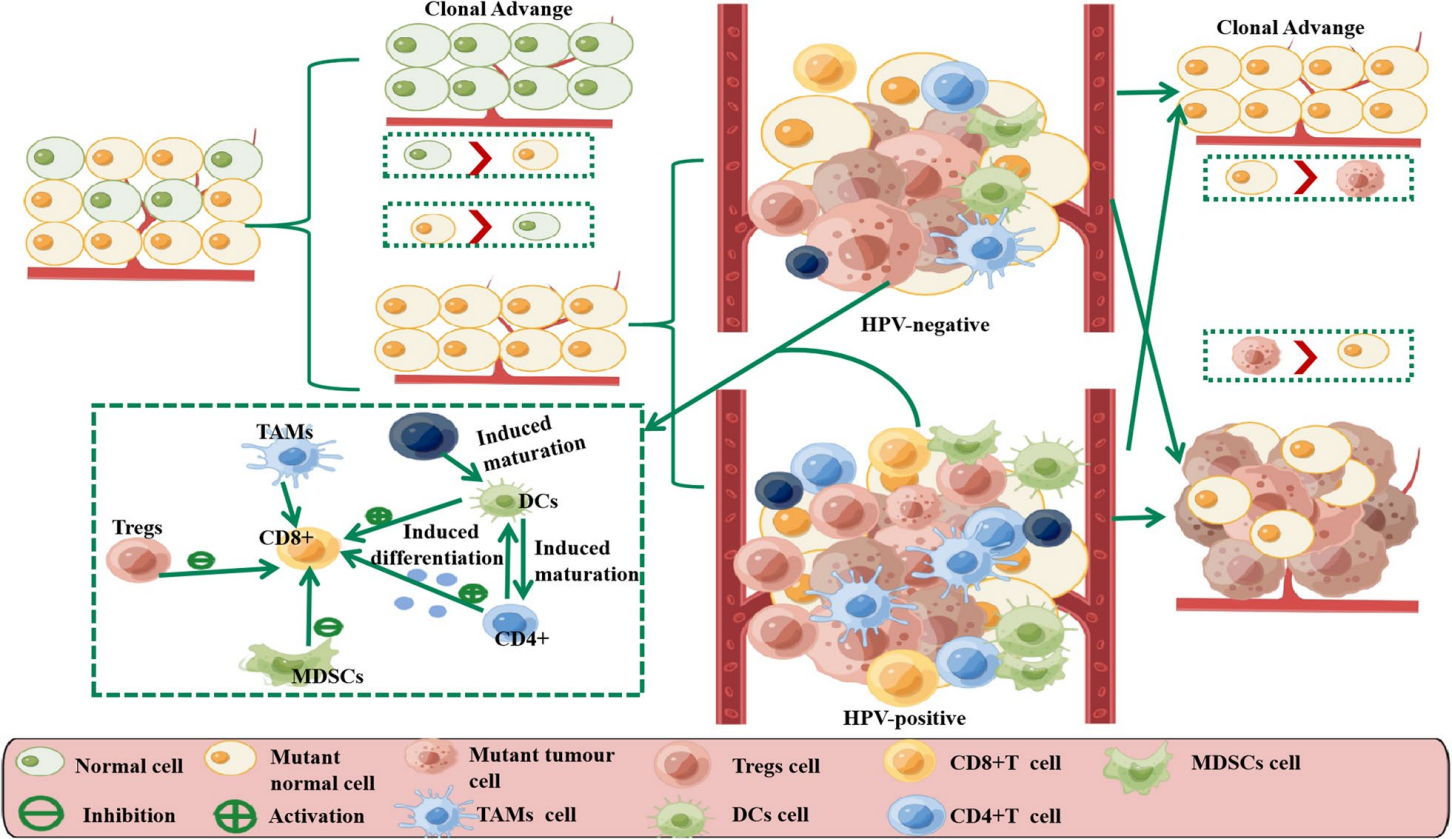
The outcome of clonal competition between HPV-positive and HPV-negative laryngeal cancers. In general, wild-type normal cells (green) are preferred over mutant cells (yellow). Nevertheless, alterations in the tumor immune microenvironment resulting from distinct etiologies (HPV-positive cell exhibiting “hot” immunity and HPV-negative cell exhibiting “cold” immunity) may grant a competitive edge to mutant cells, enabling them to surpass wild-type cells and establish dominance within the field. Consequently, mutant normal cells undergo further transformation into tumor cells (red) under the influence of the surrounding environment and subsequently remaining competitive

For LSCC, although both HPV-negative and HPV-positive LSCC are among the cancer types with the highest immune filtration rates, the degree and composition of immune cell infiltration vary depending on the etiology [81, 82] (Fig. 4). HPV-negative LSCC is characterized by a “cold” immune response, while the presence of numerous random mutations or overexpression of cellular genes contributes to intra-tumoral immune heterogeneity [83]. Reduced numbers of dendritic cells, a subpopulation of specialized antigen-presenting cells that drive T-cell differentiation, were seen in the interstitial tumor region of smoking patients. Defects in dendritic cell maturation also affect regulatory T cells as immature dendritic cells transform into gene-tolerant dendritic cells and secrete higher levels of TGF-β1, activates naive T cells to become Treg cells [84]. TGF-β expression in the larynx is reported to be higher in malignancies than in dysplastic lesions, and it may be a valuable diagnostic for malignant transformation [85]. In addition, smoking leads to less infiltration of activated cytotoxic T lymphocytes (CTLs) in intraepithelial and mesenchymal areas, thereby suppressing the immune response to TME during smoking exposure [86]. To put it another way, smoking is highly likely to weaken the immunological response mediated by T cells. T cells are critical mediators of the adaptive immune response, and an imbalanced or incorrect T cell response may contribute to cancer progression and other immune disorders. Essentially, carcinogen exposure prevents the differentiation and maturation of precursors and progenitors. Under selection pressures, proliferating cells of any differentiation stage can be susceptible to introducing and accumulating mutations, and accumulate sufficient driver mutations to get the benefits of clonal proliferation [87]. Therefore, monitoring the functional status of T cells is particularly important in HPV-negative LSCC. Furthermore, we need to understand how damage changes the tissue microenvironment and why some mutations can be both harmful and advantageous to cells depending on the tissue microenvironment.

HPV-positive LSCC has been found to exhibit a higher abundance of Tc17 lymphocytes, naive CD4 + T cells, infiltrating CD8 + T cells, bone marrow dendritic cells, and TIL cells compared to HPV-negative LSCC [82]. It has been demonstrated that a high density of tumor-infiltrating CD8 + T cells has been shown to be indicative of favorable clinical outcomes in various cancer types, including laryngeal cancer [88]. There is controversy over CD4 + T cells’ role in anti-cancer immunity, although most studies suggest that tumor-infiltrating CD4 + T cells may serve as a prognostic marker for Treg, a crucial mediator of tumor immunosuppression [89]. During the early stages of HPV infection, the expression of E5 allows the virus to evade detection by anti-viral CD4 + and CD8 + T cells. This evasion mechanism leads to increased viral persistence, replication, and spread to neighboring cells, ultimately contributing to malignant transformation [90]. Moreover, cell cultures derived from HPV-positive LSCC patients exhibit significantly elevated levels of chemokines, including CXCL21, CXCL17, CXCL12, CCL10, and CCL9, as well as slightly higher levels of cytokines such as IL-23, IL-17, IL-2, and IFN-γ. These chemokines not only play a role in establishing a pro-tumor microenvironment and facilitating organ-directed metastasis, but also contribute to disease progression [91]. HPV-positive LSCC patients showed more mDCs and slightly more pDCs and monocytes/macrophages [82]. Abundant CD68 + macrophages are related with lymph node metastases, extraperitoneal dissemination, and advanced disease [92]. All of these were attributed to the persistent expression of E6 and E7 oncoproteins, finally leading to the immune escape of tumor cells and a more malignant phenotype [77]. Overall, HPV-positive LSCC demonstrates heightened activation and infiltration of immune cells, leading to stromal alterations that exert a direct influence on the adjacent tissues, thereby facilitating the phenomenon of field cancerization and ultimately fostering the proliferation of malignant clones. Consequently, the persistent expression of the early proteins E6 and E7, in LSCC presents a promising target for immunotherapeutic interventions.

## Conclusions

An extremely common tumor in the upper aero-digestive tract, LSCC develops in a multistep process that begins with epithelial precursor lesions. This process appears to be influenced by genetic mutations, epigenetic changes, and microenvironmental changes. The occurrence of SPT in patients with LSCC is becoming increasingly common. Additionally, the clonal relationship between the two has been difficult to resolve posing a significant challenge to clinical diagnosis and treatment. So, understanding the clonal origin of cancer is critical to advancing personalized cancer treatment and reducing cancer mortality. In cases where the progenitor clone carries a genetic defect targeting a therapeutic pathway, monotherapy for cancer may be justified. However, if a patient’s tumor develops through multiclonal initiation, molecularly targeted monotherapy is unsuccessful in treating the patient. This is because the small number of untargeted clones may drive therapeutic resistance. Thus, establishing a clonal link between SPT and the index tumor is more than just a classification problem, it also provides fresh insights into the patient’s tumor biology and can influence the treatment and prognosis of the SPT. Given the unique biology of LSCC, this review thoroughly examines the clonal relationship between SPT and the index tumor in terms of the oncogenic mechanisms of the major risk factors, concluding that the HPV-negative LSCC are likely to present with dual primary independent origin LSCC/ESPT, whereas HPV-positive LSCC complicated by ESPT mostly supports the monoclonal hypothesis. In fact, the mechanisms underlying the transfer of seeds and the dynamics of evolutionary clonality in the presence of immune stress and epigenetic alterations are not yet fully understood. In future research, more in-depth exploration of the origin of tumor clones is needed to identify simpler and more accurate ways to assess the origin of clones to develop rational treatment plans and improve patient survival.
